# Miliary TB Without Microbiological Confirmation in a Post‐Menopausal Female: A Case From a High‐Burden Region

**DOI:** 10.1002/ccr3.71196

**Published:** 2025-10-08

**Authors:** Jabir Ahamad Miya, Shishir Bhandari, Pukar Adhikari

**Affiliations:** ^1^ Chitwan Medical College Teaching Hospital (CMCTH) Bharatpur Nepal

**Keywords:** computed tomography, diagnostic imaging, empirical treatment, miliary tuberculosis, Nepal, postmenopausal

## Abstract

Miliary tuberculosis (TB) presents significant diagnostic challenges in resource‐limited, high‐burden regions when microbiological confirmation is unattainable. We describe a 65‐year‐old post‐menopausal Nepalese woman with prolonged fever, anemia, and constitutional symptoms. Initial chest radiography was inconclusive, but contrast‐enhanced computed tomography (CECT) revealed diffuse miliary nodules, calcified lymphadenopathy, and pleural effusion. Microbiological confirmation was precluded by the inability to expectorate and refusal of bronchoscopy. Empirical anti‐TB therapy (isoniazid, rifampicin, pyrazinamide, and ethambutol) was initiated based on radiological findings and epidemiological context (Nepal TB prevalence: 245/100,000). The patient showed symptomatic and hematological improvement at follow‐up despite declining invasive diagnostics. This case underscores CT imaging's critical role in diagnosing miliary TB when microbiological evidence is unavailable and supports ethical empirical treatment in elderly patients from endemic regions where diagnostic limitations exist.

**Summary**

- In high TB burden regions, clinical judgement and CT imaging are critical for diagnosing miliary TB when microbiological confirmation is not possible.
- Timely empirical antitubercular therapy can be lifesaving, particularly in elderly patients unable or unwilling to undergo invasive testing.

## 1 Introduction

### 1.1 Background

Miliary tuberculosis (TB) is a disseminated form of TB caused by hematogenous spread of *Mycobacterium tuberculosis* , resulting in widespread granulomas resembling millet seeds in various organs [1]. It represents 1%–3% of TB cases and carries a high mortality rate, exceeding 25% in elderly and immunocompromised individuals [2, 3]. Diagnosis is often challenging due to nonspecific or atypical symptoms, reduced sensitivity of skin tests, and difficulty obtaining appropriate specimens for microbiological confirmation, particularly in elderly patients [4]. In clinical practice, reliance on radiological imaging and clinical judgement becomes essential.

### 1.2 Objective

We present a case of miliary TB in a post‐menopausal woman with an inconclusive chest X‐ray, no microbiological confirmation, and a diagnosis based on CT findings and clinical context.

## 2 Case History/Examination

A 65‐year‐old post‐menopausal female from Nawalpur, Nepal, presented with a 12‐day history of intermittent fever (up to 101°F), chills, anorexia, weight loss, and chest pain. She had no cough, hemoptysis, night sweats, or known TB contact. Her past medical history included hypertension, managed with Metoprolol 50 mg once daily. She recently had a UTI 2 weeks back, which was managed with oral antibiotics at a health post. She used smokeless tobacco but denied smoking or alcohol intake.

On examination, she was febrile (101.8°F), tachycardic (HR 101 bpm), normotensive (BP 110/60 mmHg), and saturating 97% on room air. Pallor was present. Fine crackles were heard in the left axillary and interscapular regions. There was no lymphadenopathy or organomegaly.

## 3 Differential Diagnosis, Investigations, and Treatment

Initial laboratory investigations revealed a hemoglobin level of 8.3 g/dL, a total leukocyte count of 7224/μL with 76.1% neutrophils, and a platelet count of 185,000/μL. C‐reactive protein (CRP) was markedly elevated at 104.9 mg/L. Liver enzymes were mildly raised, while renal function tests and blood cultures were within normal limits. On repeat testing, hemoglobin remained unchanged at 8.3 g/dL; however, there was a significant increase in total leukocyte count to 15,170/μL and a drop in platelet count to 60,000/μL. The blood culture did not show growth of any microorganisms.

**FIGURE 1:**
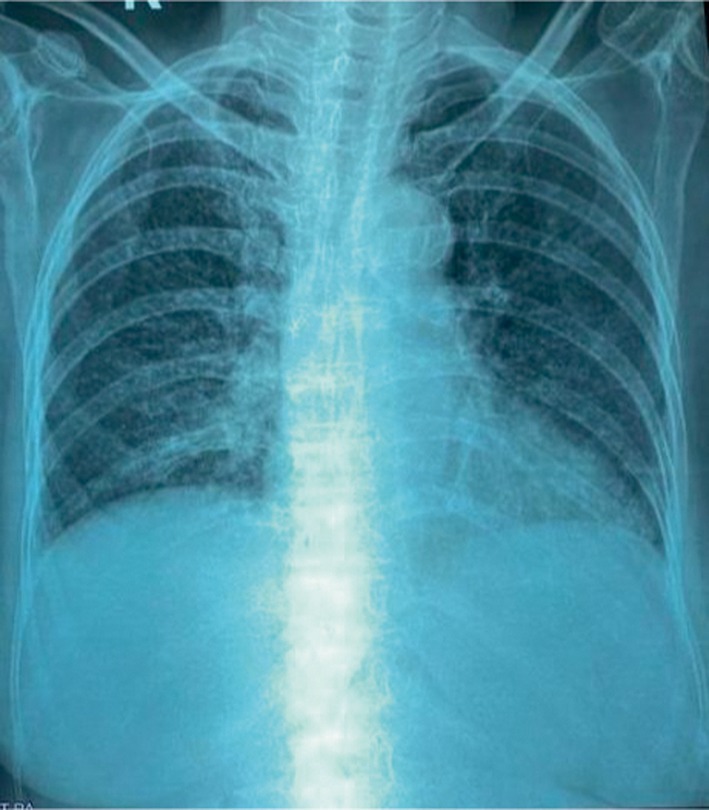
Chest X ray (PA view)—no significant findings suggestive of miliary tuberculosis.

**FIGURE 2:**
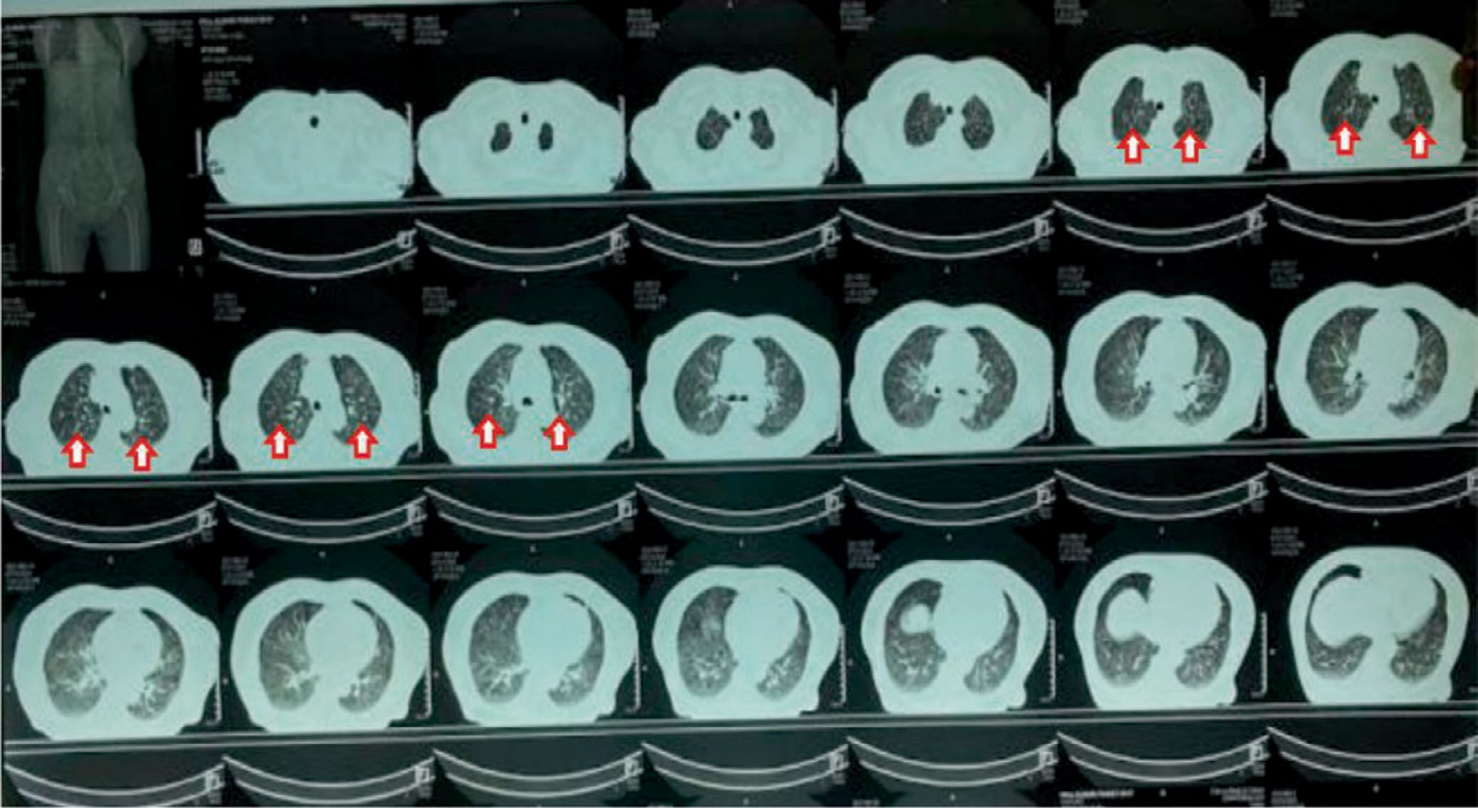
CECT chest and abdomen (axial view)—Multiple miliary nodules over bilateral lung fields (shown by red arrows).

**FIGURE 3:**
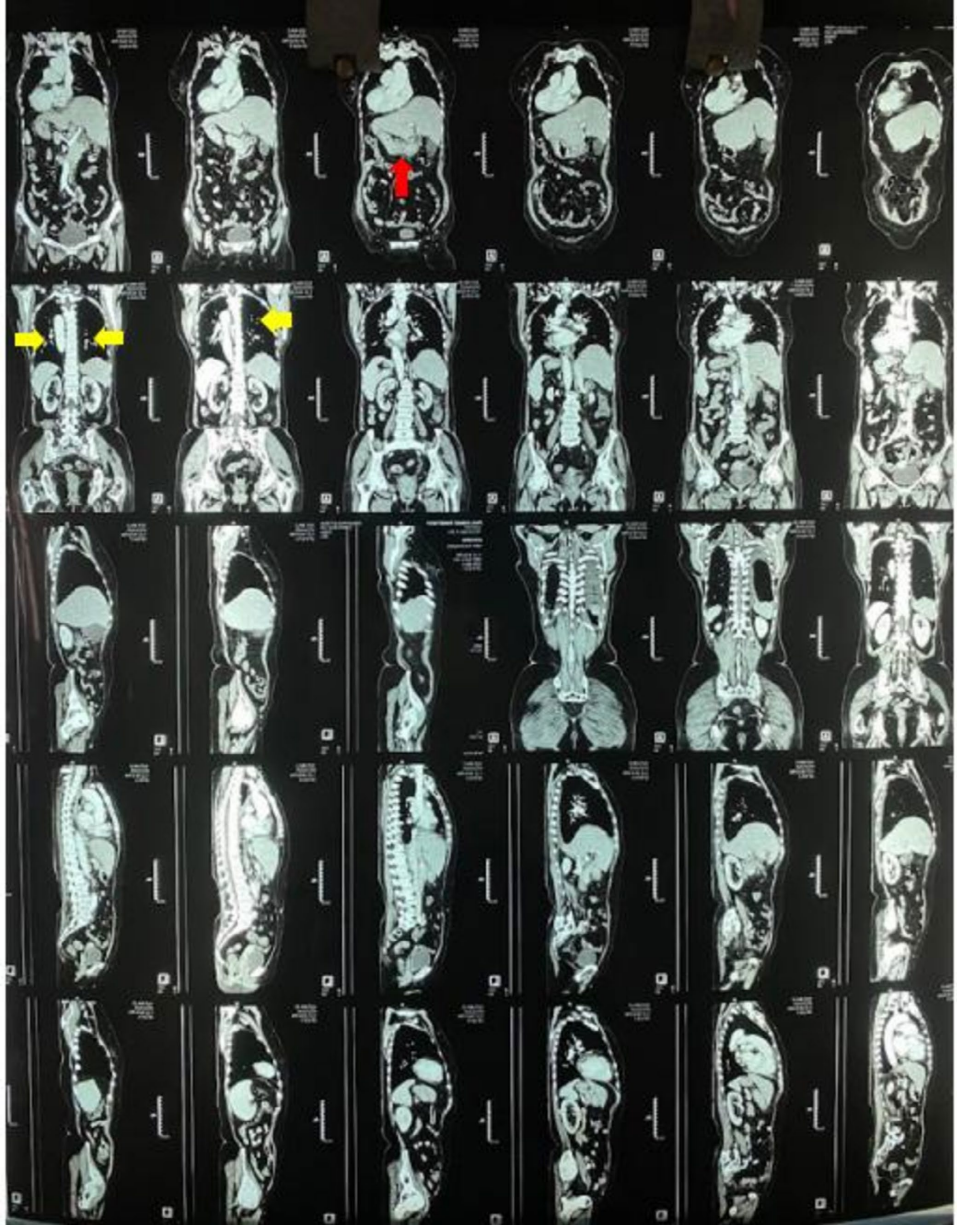
CECT chest and abdomen and (coronal and sagittal views): Miliary nodules over bilateral lung fields (yellow arrows), deformed D1 and D2 segments of duodenum (red arrow), no evidence of malignancy or metastasis in liver, spleen or kidneys.

**FIGURE 4:**
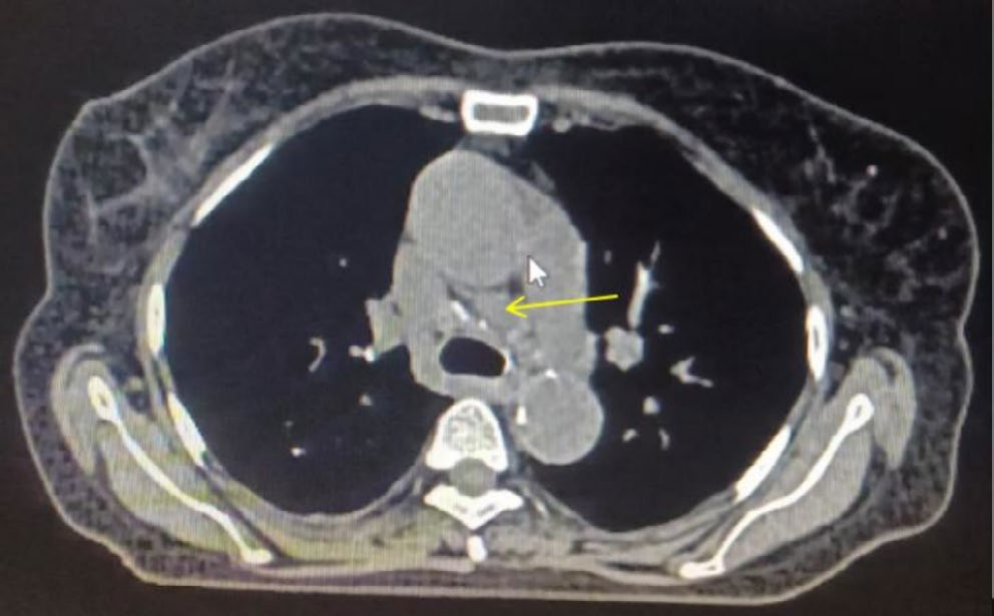
CECT chest (axial view) showing enlarged mediastinal lymph nodes (yellow arrow).

The chest X‐ray was inconclusive (Figure 1). Given persistent fever and systemic symptoms, contrast‐enhanced CT (CECT) of the chest and abdomen was performed. The findings, as shown in Figures 2, 3, 4, included multiple miliary nodules in both lungs, enlarged right paratracheal (12 × 10 mm), left paratracheal (10 × 10 mm), and subcarinal (20 × 13 mm) lymph nodes (some calcified), mild right‐sided pleural effusion, enlarged calcified mesenteric and periportal lymph nodes, a pulmonary trunk measuring 29 mm and deformed D1 and D2 segments of the duodenum without mass or thickening (suggestive of inflammatory etiology). There was no evidence of malignancy or metastasis in the liver, spleen, or kidneys. Although miliary metastasis was a differential diagnosis, the absence of a primary malignancy on systemic imaging, combined with the high TB burden in the region, favored a diagnosis of miliary TB.

Due to unexplained anemia, along with positive fecal occult blood and a history of blackish stool while admitted to the hospital, a gastroenterology consultation was sought. Upper gastrointestinal endoscopy revealed a small hiatus hernia and a lesion in the antrum characterized by an altered mucosal pattern and vascularity. No active bleeding or mass lesion was observed. Biopsies were taken from the antrum and body for *Helicobacter pylori* . A colonoscopy was also done, which showed normal findings. Microbiological confirmation of TB could not be done, as the patient was unable to expectorate. Bronchoscopy and FNAC of mediastinal lymph nodes were planned, but the patient's family declined further invasive evaluation.

Echocardiography showed moderate mitral and aortic regurgitation, mild tricuspid regurgitation, and preserved ejection fraction. Other systemic evaluations did not yield alternative explanations.

Empirical antituberculosis therapy (ATT) was initiated with a standard four‐drug regimen: isoniazid, rifampicin, pyrazinamide, and ethambutol. Prior to this, during her stay, she was managed with injectable antibiotics and other supportive medications.

## 4 Outcome and Follow Up

Despite having two documented fever spikes within 24 h before discharge, the patient showed subjective improvement and requested discharge against medical advice. She was advised to return to the medicine OPD within 3 days or earlier if needed. Sputum AFB (1 and 2), Gram stain, and GeneXpert testing were planned for follow‐up. Gastroenterology follow‐up was advised if anemia worsened. On follow‐up after 3 days of discharge (after 8 days of starting ATT), she was symptomatically better; blood workup showed an improving blood picture with hemoglobin of 9.2 g/dL, WBC count of 9800/cu.mm, and platelet count of 114,000/cu.mm. The patient's family refused invasive diagnostic tests, citing financial reasons. She was advised to continue ATT and come for follow‐up after 1 month or earlier if needed.

The discharge against medical advice limited immediate in‐hospital monitoring but did not prevent outpatient follow‐up; however, it contributed to the family's ongoing refusal of invasive diagnostics like bronchoscopy and FNAC.

Laboratory results at presentation, during hospitalization, and at follow‐up are given in Table 1.

**TABLE 1:** Laboratory findings during the course of illness.

Parameter	At presentation	During hospitalization	At follow‐up	Reference
Hemoglobin (g/dL)	8.3	8.3	9.2	12–16
Total leukocyte count (/μL)	7224	15,170	9800	4000–11,000
Neutrophils (%)	76.1	90.9	68%	40–70
Platelet count (/μL)	185,000	60,000	114,000	150,000–400,000
C‐reactive protein (mg/L)	104.9	—	—	0–5

## 5 Discussion

This case highlights critical diagnostic challenges in managing miliary TB, particularly among elderly patients in high‐burden, resource‐limited settings like Nepal [5]. While sputum smear microscopy and GeneXpert are standard diagnostic tools, their utility is limited when patients cannot produce sputum. Bronchoscopy can provide alternative samples, but it may be declined, as in this case [6]. The tuberculin skin test, not done in this case, is also often unreliable in miliary TB due to a high rate of false negatives caused by tuberculin anergy [7]. While chest X‐ray is often the first imaging modality used, it may not detect the characteristic miliary pattern, particularly in cryptic or early forms of miliary TB, underscoring its limited sensitivity (33%–67%) [8]. In contrast, a CT scan offers superior sensitivity (95%–100%) and can reveal key findings such as miliary nodules, calcified lymphadenopathy, and pleural effusion, which crucial for supporting a diagnosis of miliary TB. CT imaging also aids in excluding alternative diagnoses like miliary metastasis by assessing for primary malignancies and systemic involvement.

Gastrointestinal evaluation in this case ruled out active bleeding or malignancy, explaining anemia as part of a chronic disease or nutritional deficiency. No mass or metastasis was detected on endoscopy or CECT.

The drop in platelet count and rise in WBC during hospitalization likely reflected evolving systemic inflammation and possible bone marrow involvement—both common in disseminated TB, as supported by studies showing hematologic abnormalities in up to 50% of miliary TB cases due to granulomatous infiltration of bone marrow [1, 9, 10]. This hematologic shift further supported the clinical diagnosis.

This patient's presentation aligns with the literature on miliary TB, where nonspecific symptoms like fever, anorexia, and weight loss are common, often without classic respiratory complaints such as cough or hemoptysis, as seen in up to 30%–50% of cases in elderly patients [1, 2]. The absence of night sweats and known TB contact in this case differs from some reports but is consistent with cryptic forms described in recent case series [8]. CT imaging showed classic miliary nodules and calcified lymphadenopathy, matching high‐sensitivity CT findings in 95%–100% of confirmed cases [8], while laboratory abnormalities like anemia, thrombocytopenia, and leukocytosis reflect systemic involvement similar to those in high‐burden region studies [2]. Differential diagnoses included miliary metastasis, fungal infections, and sarcoidosis. These were deemed less likely due to the absence of a primary malignancy on CECT, endoscopy, and colonoscopy; negative blood cultures ruling out disseminated infection; and the epidemiological context of high TB incidence in Nepal (245/100,000) [11], combined with calcified lymphadenopathy more typical of TB than sarcoidosis [1, 4, 12]. Multiple poor prognostic indicators, including female gender, advanced age, thrombocytopenia, and elevated transaminases were present in this case [9]. In high TB burden regions such as Nepal, a strong index of suspicion is crucial [12, 13, 14]. Empiric anti‐TB therapy based on clinical and radiological findings is justified when microbiological confirmation is unavailable and alternative diagnoses are unlikely, as per WHO and national guidelines for high‐burden settings [15]. Evidence from high‐burden contexts also supports empirical ATT in elderly patients with atypical presentations and diagnostic challenges, where radiological findings (e.g., infiltrates, non‐cavitating lesions) and clinical suspicion outweigh the risks of delayed therapy [4]. Delayed treatment initiation can result in significant morbidity and mortality, especially in elderly patients with nonspecific presentations.

## 6 Conclusion

The diagnosis of miliary TB may be challenging without microbiological confirmation, especially in elderly patients unable or unwilling to undergo invasive testing. This case highlights the vital role of clinical suspicion, CT imaging, and exclusion of alternate causes, including malignancy through endoscopy, in guiding timely empirical treatment. Given miliary TB's high mortality risk in untreated patients, clinicians must prioritize therapeutic intervention over diagnostic delay when clinical and radiological evidence strongly supports disseminated disease.

## Author Contributions

**Jabir Ahamad Miya:** conceptualization, data curation, visualization, writing – original draft, writing – review and editing. **Shishir Bhandari:** data curation, investigation, methodology, visualization, writing – review and editing. **Pukar Adhikari:** project administration, supervision, validation, writing – review and editing.

## Ethics Statement

Ethical approval was waived per institutional guidelines for de‐identified case reports.

## Consent

Written informed consent was obtained from the patient for publication of this case report and accompanying images. A copy of the written consent is available for review by the Editor‐in‐Chief of this journal on request.

## Conflicts of Interest

The authors declare no conflicts of interest.

## Data Availability

No datasets were generated or analyzed during the preparation of this manuscript.
